# On the Relationship between Reading Abilities and Word Properties Involved in Word Recognition

**DOI:** 10.5334/joc.484

**Published:** 2026-01-12

**Authors:** Daniele Gatti, Davide Crepaldi, Serena Lecce, Luca Rinaldi, Sara Mascheretti

**Affiliations:** 1 Department of Medicine and Surgery, University of Parma, Parma, Italy; 2 Department of Brain and Behavioral Sciences, University of Pavia, Pavia, Italy; 3 Cognitive Psychology Unit, IRCCS Mondino Foundation, Pavia, Italy; 4 Child Psychopathology Unit, Scientific Institute IRCCS Eugenio Medea, Bosisio Parini, Italy

**Keywords:** reading, lexical decision, length, frequency, semantics

## Abstract

Word recognition is a complex cognitive process that has been often investigated via lexical decision task (LDT). LDT can indeed provide insight into how individuals access and process linguistic information, and how (and if) specific word- and/or individual-level characteristics affect participants’ behavior. Here, we aimed to provide a systematic investigation of the interaction between individual-level reading skills and word-level factors (e.g., frequency, length). Participants were asked to perform a LDT and complete neuropsychological tests assessing their reading-related skills. By using completely data-driven approaches, participants’ performance in the LDT was predicted by word- and individual-level predictors, and the best-fitting model was selected. The best-fitting model dropped all the interactions among deeper-level predictors (e.g., density of the semantic neighborhood) and reading-related skills. The interactions involving word length or word frequency indicated that more expert readers are less sensitive to this kind of factors. These results underscore the importance of considering both lexical properties and individual reading proficiency when investigating the cognitive mechanisms underlying word recognition.

## Introduction

Word recognition is a complex cognitive process that relies on multiple perceptual, attentive, lexical, orthographic and semantic components. Previous works investigating these processes employed lexical decision tasks (LDT), in which participants are asked to judge whether a given string of letters is a word or not (Balota et al., 2007; Keuleers et al., 2012). This task provides insight into how individuals access and process linguistic information, and how (and if) specific word characteristics affect participants’ behavior, such as response times.

Well-known word characteristics playing a role in the LDT are, for example, word length (generally indexed as number of characters or syllables) and word frequency (i.e., number of appearances of a target word in a given corpus). These two effects typically have opposite directions; the longer a word, the slower the RT, while higher frequency would normally yield quicker responses (e.g., Balota et al., 2004; Spieler & Balota, 1997). Length effects are often attributed to the increased processing demands associated with longer letter strings, particularly in tasks requiring sublexical processing (Yap & Balota, 2009). On the other hand, word frequency effects are typically explained through models of lexical access that posit faster processing for more familiar words (Brysbaert et al., 2018).

Other word characteristics that come into play in the LDT involve orthographic and semantic processing. Several studies demonstrated that words with a denser (semantic or orthographic) neighborhood (i.e., words that have comparatively more similar words, either semantically or orthographically) are recognized faster (e.g., Hendrix & Sun, 2021; Anceresi et al., 2025), as well as those with a higher orthographic-to-semantic consistency (i.e., the extent to which orthographically similar words share similar meanings; Marelli et al., 2015; Marelli & Amenta, 2018).

Besides word-level components, several studies investigated individual-level predictors of humans’ response times in the LDT (e.g., Ferré et al., 2024; Haro et al., 2024). Notably, reading skills have been identified as one of the major individual-level components of interest. Previous studies have showed that age-related declines in the LDT performance are moderated by better reading skills (Lee & Kim, 2024), and that expert readers or individuals possessing better vocabulary knowledge exhibit faster and more accurate responses (Balota et al., 2007). Other studies have focused on the interaction between reading skills and some of the word-level superficial characteristics, showing that length effects are more pronounced in individuals with developmental dyslexia (DD) compared to typical readers (e.g., Araújo et al., 2014; Juphard et al., 2004; Martens & de Jong, 2006). However, previous studies implemented a dichotomous approach to reading skills (e.g., fast *versus* slow readers, typical readers *vs*. individuals with DD), thus neglecting the investigation of the effects along the entire inter-individual variability spectrum (i.e., including reading skills as a continuous factor). In addition, despite the growing interest in semantic and orthographic processes, a systematic investigation of the effects of both superficial- and deeper-level components (and their potential interaction with reading skills) is missing.

In the present study, we addressed this gap by examining the interaction effects between various reading-related skills (i.e., reading accuracy and speed, non-verbal cognitive skills, working memory, and cross-modal mapping proficiency) and commonly studied word features in LDTs that index a various set of components (e.g., length, frequency, density of the semantic neighborhood). Our hypotheses were built on seminal evidence on reading acquisition indicating that more proficient readers process progressively larger chunks (Andrews & Hersch, 2010; Andrews & Lo, 2013). Accordingly, more proficient readers should rely less on components such as word length as compared to deeper, semantic ones (e.g., on the amount of semantic information carried by larger chunks). We thus expected a specific pattern of results, with more proficient readers benefiting most from semantic components (e.g., stronger semantic effects), while relying less on more superficial ones (e.g., smaller length effects).

## Method

### Participants

One-hundred and five students participated in the study (80 females, *M* age = 22.30 years, *SD* = 2.14). All participants were native Italian speakers, had normal or corrected to normal vision and were naïve to the purpose of the study. Informed consent was obtained from all participants before the experiment. The protocol was approved by the ethical committee of the University of Pavia and participants were treated in accordance with the Declaration of Helsinki. Sample size was estimated a priori aiming to test at least 100 participants as it is common for individual differences studies (Brysbaert, 2019).

### Lexical decision task

The words included in the study were selected among the 20,000 most frequent Italian words according to the SUBTLEX-it (https://osf.io/zg7sc/), while the pseudowords were selected among a large set of around 100,000 stimuli built using the orthographic Italian module of Wuggy (Keuleers, & Brysbaert, 2010) starting from the words included in the Italian ANEW database (Montefinese et al., 2014). From the generated pool, we selected 100 pseudowords that were orthotactically legal and had length and orthographic neighborhood density comparable to the word stimuli (BFs > 5 in favor of the null hypothesis, JASP independent t-test). This helps avoiding that lexical decision difficulty was driven by trivial low-level stimulus differences between words and pseudowords. The final set included 200 stimuli (100 words and 100 pseudowords).

Words were selected to have a quasi-uniform distribution of our main semantic predictors of interest (semantic neighborhood density – SND and orthographic-to-semantic consistency – OSC) and, at the same time, not to have unbalanced distributions of the other predictors included (i.e., orthographic neighborhood density – OND, length, and frequency). For details see Supplementary Material.

For each word we computed length as number of characters, we retrieved word frequency from the SUBTLEX-it and computed the mean bigram frequency (MBF) as the average (log-transformed) frequency-weighted count of a word’s letter bigrams in the lexicon (considering the SUBTLEX-it as the lexicon).

The semantic predictors were both computed following Hendrix & Sun (2021; but see also Anceresi et al., 2025; and Marelli & Amenta, 2018). SND was defined as the average semantic similarity between each word and its *k* closest semantic neighbors (with *k* = 5) within the 20,000 most frequent words in SUBTLEX-it. Higher values thus indicate that a certain stimulus is more embedded in the semantic space. OSC was defined as the average semantic similarity between each word and its *k* closest orthographic neighbors (in terms of Levenshtein distance, see below OND; with *k* = 5) within the 20,000 most frequent words in SUBTLEX-it. Higher values thus indicate higher consistency between form and meaning. For more information on the computation of the semantic similarity see the “Distributional semantic model” paragraph.

OND was computed as the average Levenshtein distance (which measures the orthographic distance between two strings of symbols by quantifying the minimum number of single-character edits required to change one element into the other) between the letter string and its 20 closest orthographic neighbors (i.e., OLD20; Yarkoni et al., 2008) among the 20,000 most frequent words in SUBTLEX-it. Length was computed as the number of letters in the letter string, and word frequency was retrieved from the SUBTLEX-it and log-transformed to account for its skewed distribution (as common in this kind of tasks Hendrix & Sun, 2021). Distributions of and correlations among the predictors are reported in Figure SM1.

The task was built using Psychopy (Peirce et al., 2019). Participants were shown one word at the time and were asked to indicate if the stimulus was an Italian word or not as fast and accurately as possible by pressing the ‘A’ or ‘L’ key using the left and right index fingers, respectively. The trials were presented in random order. Each trial started with a central fixation cross (presented for 500 ms) and was followed by a string of letters (presented for maximum 5000 ms). Participants’ response ended the trial and moved to a blank screen (presented for 1000 ms), which preceded the fixation cross of the next trial.

### Reading measures

Participants underwent two reading tests from the “LSC-SUA: Battery for the Assessment of Learning Disabilities and Other Disorders in University Students and Adults” (Montesano et al., 2020).

More specifically, the tests administered here assess word and pseudo-word (strings of pronounceable but non-existent words in the Italian language) reading abilities. The word reading test requires participants to read aloud four lists each containing 28 words of different length and frequency of use. The pseudo-word reading test involves reading aloud two lists each containing 28 pseudo-words of different lengths. Accuracy (number of errors) and speed (time) were assessed for both tests. Norms were available for adults from 18 to 66 years; for this study, we referred to norms for 18-to-35-year subjects. These tests showed large effect sizes (word reading speed: Cohen’s d = 2.58, 95% CI = 0.84–0.95; word reading accuracy: Cohen’s d = 1.72, 95% CI = 0.77–0.89; pseudo-word reading speed: Cohen’s d = 1.91, 95% CI = 0.79–0.92; pseudo-word reading accuracy: Cohen’s d = 1.47, 95% CI = 0.70–0.83) in discriminating subjects with reading impairments and typical readers (Montesano et al., 2020). The administered Italian version was standardized on a sample of 875 participants attending different Italian universities and was representative of the general population in terms of sex and socio-economic background distributions (Montesano et al., 2020). Favoring the ability of this instrument of capturing nuances in reading abilities, previous data showed that the observed participants’ performance was continuous and not dichotomic (i.e., it goes beyond a mere poor-good reader classification; Montesano et al., 2020).

### Additional Neuropsychological Measures

Additional neuropsychological skills were assessed in all the participants using the following tasks: i) the Cattell’s Fluid Intelligence Test (Cattell, 1949); ii) the Digit Span subtest from the Wechsler Adult Intelligence Scale (Wechsler, 2008) to assess working memory (WM); and, iii) the serial rapid automatized naming (RAN) task (De Luca et al., 2005). For details see Supplementary Material.

### Distributional semantic model

The DSM used was *fastText* (Joulin et al., 2016, for a review: Bonandrini & Gatti, 2024), and word vectors were retrieved from the Italian pretrained vectors (Grave et al., 2018). The model was trained on Italian Wikipedia using the skip-gram method with 300 dimensions, character n-grams of a length of 3- to 6-, and a window of size 5. With respect to traditional distributional models, whose ability to generate high-quality distributed semantic representations is limited to words that are sufficiently frequent in the input data, fastText is based on the idea (originally proposed by Schutze, 1992; and realized by Bojanowski et al., 2017) to take into account sub-word information by computing word vectors as the sum of the semantic vectors for the n-grams associated with each word.

Using *fastText*, we therefore obtained semantic representations for the words included in this study as well as for the 20,000 most frequent words included in the SUBTLEX-it that were used to compute SND and OSC indexes, with semantic similarity being indexed as the cosine of the angle formed by vectors representing the meanings of the corresponding strings. The higher the cosine value, the more semantically related the letter strings are expected to be, as estimated by the model.

### Procedure

Participants were tested individually at the University of Pavia, in a quiet room. They were firstly asked to complete the lexical decision task followed by word and pseudo-word reading, non-verbal cognitive, WM, and RAN tests.

## Data analysis and results

As mean bivariate correlations (*r*) were moderate among the administered neuropsychological tests (*r* = .488, see Figure SM2 for distributions and correlations among the variables), we ran a Principal Component Analysis (PCA). This allowed us to find the optimal weights for the variables to account for the maximum amount of variance in the dataset, with the smallest number of underlying factors (Norman & Streiner, 2008). We considered the following (standardized) parameters: i) word reading speed and accuracy; ii) pseudo-words reading speed and accuracy; iii) digits span performance; and, iv) colors and objects naming. Using a promax rotation method, we obtained two factors with an eigenvalue > 1.0, explaining 55.01% and 19.02% of the total variance, respectively (Kaiser-Meyer-Olkin measure of sample adequacy = .727, Bartlett test of sphericity, *χ^2^*(21) = 440.026, *p* < .001). Due to the parameters composing the two factors, we respectively labeled them as ‘automatization’ and ‘reading accuracy’. In order to determine which variable loads on which factor, we applied a 5% level of significance settling loadings’ value to .386 (Norman & Streiner, 2008; Table 1). Standardized regression scores were then saved for each subject and entered as behavioral predictors in subsequent analyses.

**Table 1:** Rotated Component Matrix (extraction method: principal component analysis; rotation method: promax).


	COMPONENTS	

	AUTOMATIZATION	READING ACCURACY

**Word reading speed**	**0.829**	0.114

**Word reading accuracy**	–0.017	**0.902**

**Pseudoword reading speed**	**0.740**	0.097

**Pseudoword reading accuracy**	–0.050	**0.918**

**WM**	**0.479**	0.377

**RAN colors**	**–0.950**	0.073

**RAN objects**	**–0.959**	0.181


WM = Working memory; RAN = Rapid automatized naming.

Data from the lexical decision task was analyzed through a mixed-effects approach (i.e., Linear mixed models – LMMs), which incorporates both fixed-effects and random-effects (associated to participants and items) and allows for managing non-independency of the observations at both participants and item level (Baayen, 2008). Linear mixed models (LMMs) were estimated using the *lme4 R* package (Bates, et al., 2015).

The dependent variable was participants’ correct response times (RTs) for the included words, which were log-transformed to account for their natural positively skewed distribution (see Gatti et al., 2023). The LMM included the two predictors resulting from the PCA (i.e., automatization and reading accuracy) at the individual level as continuous predictors; SND, OSC, OND, word frequency, length and MBF, at the item level; and, their interactions with the individual-level predictors. Participants and items were included as random intercepts. More specifically, the model estimated was:


\[
\begin{array}{*{35}{l}} {\rm logRTs} ~ \sim ~\left({\rm SND}+{\rm OSC}+{\rm OND}+{\rm word}\_{\rm frequency}+{\rm length}+{\rm MBF}\right) \\ \times ~~\left({\rm automatization}+{\rm reading} ~{\rm accuracy}\right)+\left(1 |{\rm ID}\right)+\left(1|{\rm item}\right) \\\end{array}\]

We then performed a model selection using the *MuMIn R* package, with the function *dredge* (Bartoń, 2020). This procedure selects the best fitting model (i.e., the one with the lowest Akaike information criterion (AIC), which returns an estimation of the quality of the model; Akaike, 1973) after fitting all the possible combinations of the fixed effects included. The best model was selected as the one with i) the lowest AIC, and ii) Δ AIC > 2 with all the other models (Hilbe, 2011). If it was not possible to meet both these criteria, we then selected as best model the one i) being more parsimonious (i.e., the one with less parameters) among the ones with the lowest AIC, and ii) having Δ AIC > 2 with the other more parsimonious models.

Participants’ accuracy was very high. All the participants had accuracy proportion > .90, with a mean accuracy proportion = .98 ± .02. Trials in which participants incorrectly classified the word as a pseudo-word or in which RTs were shorter than 300 ms or longer than 3000 ms were removed from the analysis (2.2% of the trials removed).

The best model (selected *via* parsimony) was:


\[
\begin{array}{*{35}{l}} {\rm logRTs} ~ \sim {\rm OSC}+{\rm word}\_{\rm frequency}+{\rm length}+{\rm automatization}+{\rm reading} ~{\rm accuracy} \\ +~{\rm automatization:}~~{\rm length}+{\rm reading} ~{\rm accuracy:}~~{\rm word}\_{\rm frequency}+\left(1 |{\rm ID}\right)+\left(1|{\rm item}\right) \\\end{array}\]

In other words, the best model included OSC, word frequency, length, automatization, reading accuracy, and the interactions between automatization and length and between reading accuracy and frequency (*Pseudo-R² (marginal)* = .19, *Pseudo-R² (total)* = .53; Table 2). All the other fixed factors were dropped.

**Table 2:** Results of the best-fitting model resulting from the model selection procedure.


FIXED EFFECT	*F*-VALUE	*NumDF, DenDF*	*p*-VALUE	*b*

OSC	17.55	1,94	**< .001**	**–.19**

word frequency	30.73	1,94	**< .001**	**–.02**

length	75.43	1,94	**< .001**	**.04**

automatization	1.40	1,137	.23	–.03

reading accuracy	2.03	1,133	.15	–.03

automatization : length	85.88	1,10049	**< .001**	**–.01**

reading accuracy : word frequency	8.44	1,10049	**.003**	**.003**


**Figure 1:**
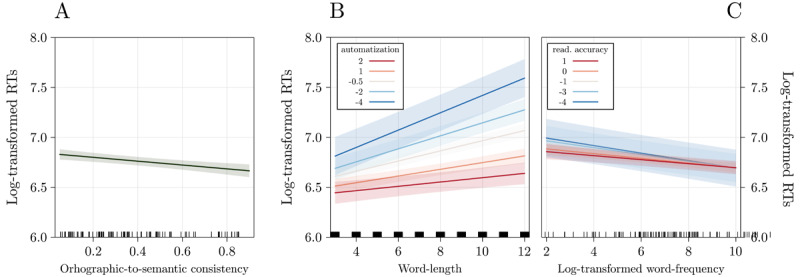
Results of the best-fitting model on participants’ RTs. RTs decreased as a function of OSC **(A)**; the interactions automatization by length and reading accuracy by word frequency indicated that expert readers (in warmer colors) are less sensitive to the length effect **(B)** and to the word frequency effect **(C)**.

**Figure 2:**
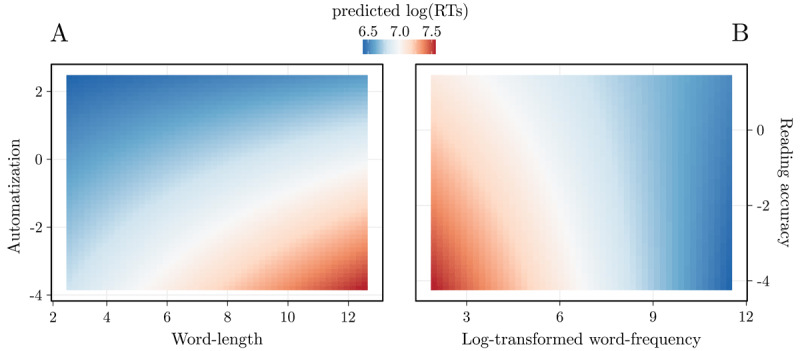
Contour plots of the interactions automatization by length **(A)** and reading accuracy by frequency **(B)**. The individual-level predictor is on the Y-axis, the word-level predictor is on the X-axis, while the timing of the RTs is indicated by the color populating both plots, with warmer colors indicating slower RTs. The lower sensitivity to length and frequency effects showed by more expert readers can be understood by comparing the colors spectrum from the upper (low variability) to the lower (high variability) parts of the plots.

More specifically, the negative effect of OSC indicates that the higher the form-meaning consistency, the faster the participants’ responses (Figure 1A). From a visual inspection, we can infer that i) the significant interaction between automatization and length indicates that the longer the word, the slower the participants are in classifying it, with participants having higher automatization scores being less affected by this component (Figures 1B and 2A); and, ii) the significant interaction between reading accuracy and word frequency indicates that the more frequent the word, the faster the participants are in classifying it, with participants having higher reading accuracy scores being less affected by this component (Figures 1C and 2B).

## Discussion

In the present study we investigated the relationship between individual-level, reading-related skills and both various word properties involved in word recognition. Participants were asked to perform a lexical decision task (LDT) and to complete neuropsychological tests assessing their reading-related proficiency. By using a completely data-driven approach, we identified two main factors accounting for the maximum amount of variance in individual-level measures and used them to predict participants’ behavior in the LDT. The two main factors (i.e., automatization and reading accuracy) were used to predict participants’ latencies in interaction with common word-level indices typically studied in LDTs indexing components such as length, word frequency or density of the semantic neighborhood. The best-fitting model explaining our data excluded all the interactions among semantic components and reading-related skills, while retaining those interactions involving length and word frequency, which were in the expected direction.

Overall, the finding that more expert readers are less sensitive to superficial-level components aligns with previous research showing that less-skilled readers, such as individuals with DD, could tend to rely more heavily on sublexical information (in this case indexed by word length), which would make them more sensitive to superficial-level word characteristics (Araújo et al., 2014; Martens & de Jong, 2006; Juphard et al., 2004). Similarly, the observed interaction between reading accuracy and word frequency indicates that expert readers are less influenced by word familiarity, possibly because they are more proficient at processing even low-frequency items efficiently. We can advance two-fold interpretation for this pattern. On the theoretical side, it is possible that individuals with higher reading-related skills have a more efficient lexical route, thus supporting seminal models assuming the existence of a lexicon (e.g., Beyersmann & Grainger 2023; Coltheart et al., 2001; Crepaldi et al. 2010; Grainger & Ziegler 2011; McClelland & Rumelhart, 1981). On the practical side, the word frequency estimates used in the current study could not actually index word familiarity for all the participants at the same level, with more expert readers being more familiar with more (or different) words than less expert ones. This would in turn suggest the need to tailor certain word-level predictors at the individual-level.

On the other hand, the predicted interaction between semantic characteristics and reading abilities was not observed for any of the predictors considered. That is, although orthographic-to-semantic consistency was included in the best fitting model suggesting that form-meaning consistency facilitates recognition across participants (Marelli et al., 2015; Marelli & Amenta, 2018), its effect did not vary as a function of individual reading skills. It is possible that the depth of the processing required by the LDT is relatively shallow and may not sufficiently tap into the semantic access variability that would be heavier in tasks requiring richer contextual integration, such as sentence reading or comprehension tasks.

Overall, these findings indicate that expert readers can more effectively bypass length or word frequency processes and engage in faster, more direct lexical access (see, for example, Grainger and Ziegler, 2011). At the same time, the lack of modulation for semantic variables raises questions about the degree to which individual differences in reading abilities influence deeper semantic processing in LDTs (but cfr. Anceresi et al., 2025 for evidence for sighted vs. blind individuals’ different reliance on semantic processing in this type of task).

Framing our findings within a diffusion-like perspective, lexical decision times likely reflect multiple components, including stimulus encoding and motor execution (often grouped as non-decision time), the rate at which lexical/semantic evidence accumulates, and individual decision caution (e.g., Ratcliff et al., 2004). Within this perspective, length may primarily increase early orthographic/encoding demands, whereas word frequency (and OSC) may change the efficiency of lexical access and the quality of form-meaning evidence. Individual differences in reading-related skill may therefore modulate different decisional components (e.g., faster/cleaner accumulation for difficult items, reduced sensitivity to encoding costs, or different caution settings), offering an interesting account for the processes at hand.

In conclusion, this study further contributes to the understanding of how reading abilities shape word recognition processes. While results show that expert readers are less affected by word length and word frequency, as they rely more on parallel, automatic orthographic processing, the role of individual differences in reading proficiency and its possible role as a moderator of deeper semantic effects remains less clear. These results underscore the importance of considering both lexical properties and individual reading skills when investigating the cognitive mechanisms underlying visual word recognition.

## Data Accessibility Statement

All data, scripts and codes used in the analysis are available at: https://osf.io/tu36g/?view_only=28c4d0b6abd14c2292b6dba969b2e99d.

This study was not preregistered.

## Additional File

The additional file for this article can be found as follows:

10.5334/joc.484.s1 Supplementary Material. Figures SM1 and SM2.
