# Supplementary material for: On the Relationship between Reading Abilities and Word Properties Involved in Word Recognition
Source: J Cogn. 2026 Jan 12;9(1):11. doi: 10.5334/joc.484 (PMC12802098; doi:10.5334/joc.484)
Supplement: Supplementary Material. — Figures SM1 and SM2. [file joc-9-1-484-s1.pdf]

Supplementary Material

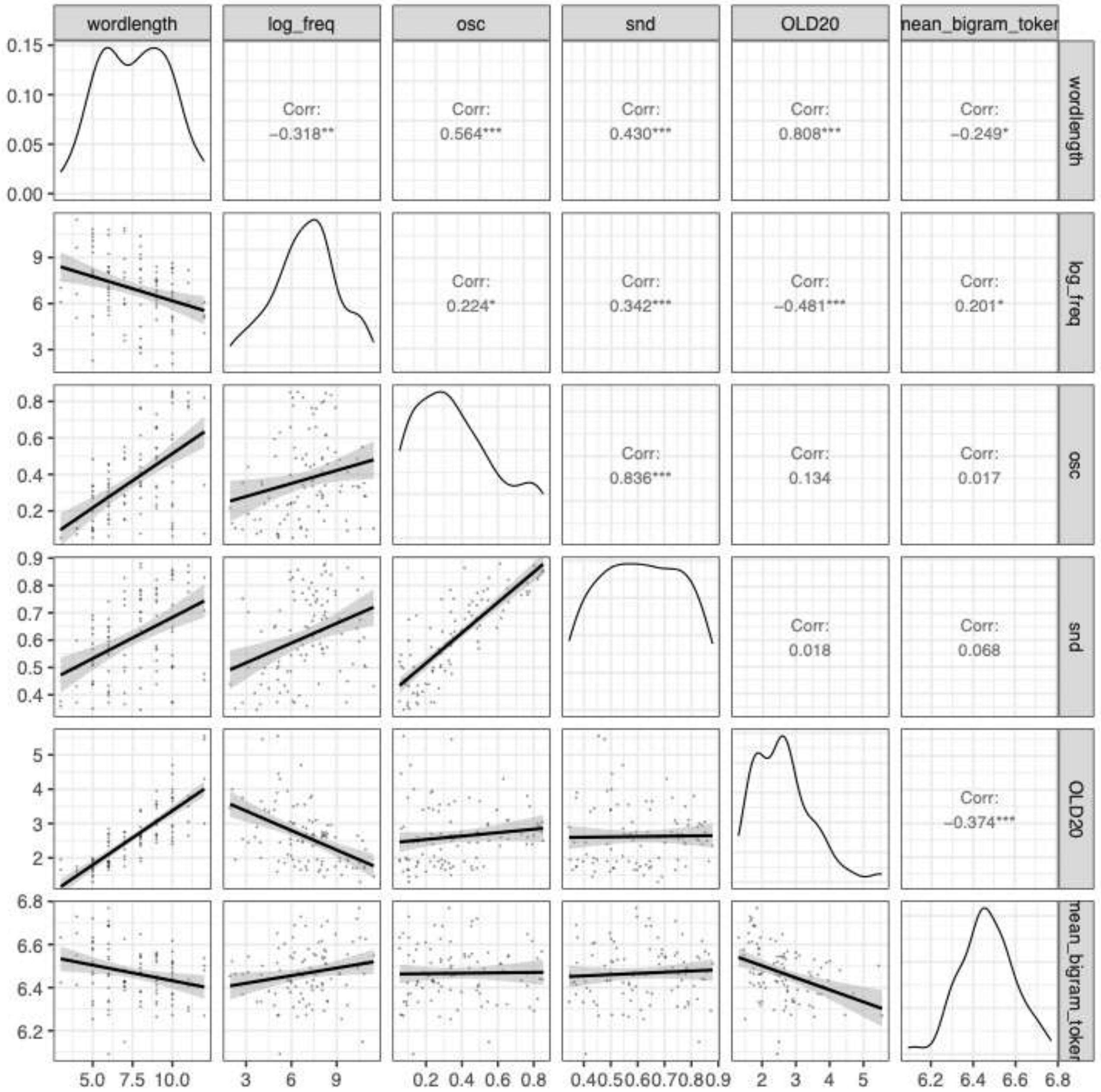

Figure SM1. Correlation matrix of the word-level predictors considered.

(Complete description of) Additional Neuropsychological Measures

Non-verbal cognitive skills. The Cattell's Fluid Intelligence Test (Cattell, 1949) was used to assess this domain (internal consistency: .80 < Cronbach α < .90; concurrent validity: good correlation with other intelligence tests; Cattell, 1949). It comprises four subtests: Series, Classification, Matrices, and Analogies. In the Series subtest, participants must complete a sequence of figures by selecting the correct missing figure from five options. The Classification subtest requires

identifying the odd figure in a group. In the Matrices subtest, participants must complete a matrix by choosing the correct missing part from five options. In the Analogies subtest, participants must select, from five options, the figure that correctly corresponds to a reference figure. Since this is a non-verbal intelligence test, it is suitable for measuring intelligence independently of verbal fluency, cultural background, and educational level. Norms were available for subjects from 7 years to adults; for this study, we referred to norms available for adults.

Working memory. The Digit Span subtest from the Wechsler Adult Intelligence Scale – Fourth edition (Wechsler, 2008) was administered (internal consistency: Cronbach α ~ .80; good test-retest stability; Wechsler, 2008). It consists of three tasks (i.e., forward span, backward span, and digit sequencing). Each task includes two sets of eight items of increasing difficulty (ranging from 2 to 9 digits for the forward span and digit sequencing, and from 2 to 8 digits for the backward span). In each item, participants hear a numerical sequence and are asked to repeat it in the same order (forward span), in reverse order (backward span), or in ascending order (digit sequencing). Norms were available from subjects aged from 16 to 90 years; we here referred to norms for 18-to-29-year participants.

Cross-modal mapping. Cross-modal mapping from visual stimuli to the correspondent spoken words as fast and accurate as possible (Denckla and Rudel, 1976) was measured by using a serial rapid automatized naming (RAN) task (De Luca et al., 2005). Extensive research has provided reliable findings supporting RAN as one of the best longitudinal predictors of reading fluency across all orthographies (e.g., reliability and validity > .90; Howe et al., 2006; Norton & Wolf, 2012). In the current study, we used RAN for non-alphanumerical stimuli (i.e., colors and objects). Both tests consist of a matrix 4x5 preliminary items and two matrices 10x5 stimuli. Accuracy (number of errors) and speed (time) were assessed for both tasks. For this study, we focused on speed as a ceiling effect was observed for accuracy. Standardized scores were estimated according to our sample's distribution.

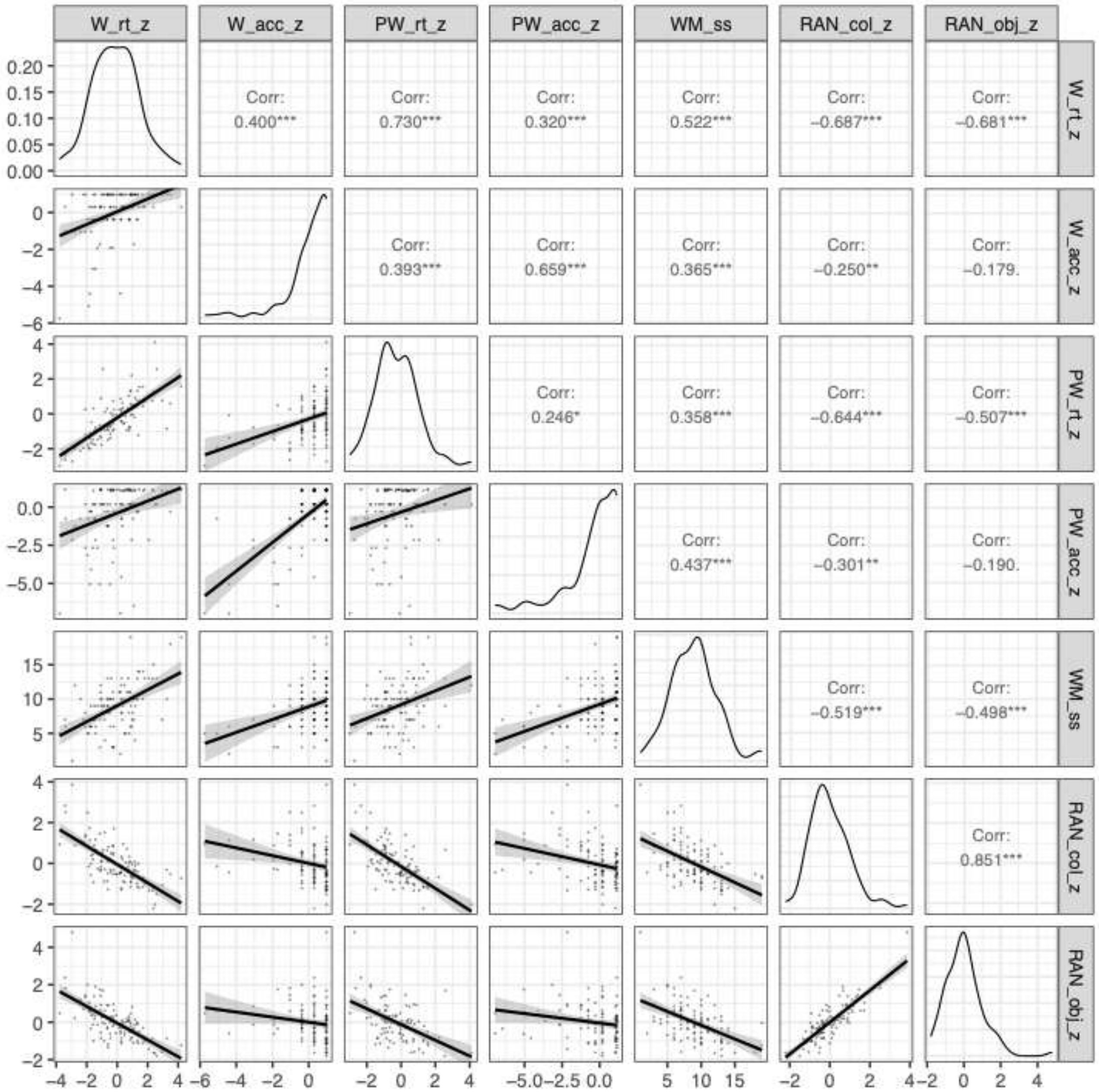

Figure SM2. Correlation matrix of the individual-level predictors included in the PCA.
